# Prevalence and onset of comorbidities in the CDKL5 disorder differ from Rett syndrome

**DOI:** 10.1186/s13023-016-0418-y

**Published:** 2016-04-14

**Authors:** Meghana Mangatt, Kingsley Wong, Barbara Anderson, Amy Epstein, Stuart Hodgetts, Helen Leonard, Jenny Downs

**Affiliations:** Telethon Kids Institute, The University of Western Australia, Perth, WA Australia; School of Anatomy, Physiology & Human Biology, the University of Western Australia, Perth, WA Australia; Western Australian Neuroscience Research Institute, Perth, WA Australia; School of Physiotherapy and Exercise Science, Curtin University, Perth, WA Australia

**Keywords:** CDKL5 disorder, Rett syndrome, Comorbidities, Cyclin dependent kinase-like 5 gene, MECP2

## Abstract

**Background:**

Initially described as an early onset seizure variant of Rett syndrome, the CDKL5 disorder is now considered as an independent entity. However, little is currently known about the full spectrum of comorbidities that affect these patients and available literature is limited to small case series. This study aimed to use a large international sample to examine the prevalence in this disorder of comorbidities of epilepsy, gastrointestinal problems including feeding difficulties, sleep and respiratory problems and scoliosis and their relationships with age and genotype. Prevalence and onset were also compared with those occurring in Rett syndrome.

**Methods:**

Data for the CDKL5 disorder and Rett syndrome were sourced from the International CDKL5 Disorder Database (ICDD), InterRett and the Australian Rett syndrome Database (ARSD). Logistic regression (multivariate and univariate) was used to analyse the relationships between age group, mutation type and the prevalence of various comorbidities. Binary longitudinal data from the ARSD and the equivalent cross-sectional data from ICDD were examined using generalized linear models with generalized estimating equations. The Kaplan-Meier method was used to estimate the failure function for the two disorders and the log-rank test was used to compare the two functions.

**Results:**

The likelihood of experiencing epilepsy, GI problems, respiratory problems, and scoliosis in the CDKL5 disorder increased with age and males were more vulnerable to respiratory and sleep problems than females. We did not identify any statistically significant relationships between mutation group and prevalence of comorbidities. Epilepsy, GI problems and sleep abnormalities were more common in the CDKL5 disorder than in Rett syndrome whilst scoliosis and respiratory problems were less prevalent.

**Conclusion:**

This study captured a much clearer picture of the CDKL5 disorder than previously possible using the largest sample available to date. There were differences in the presentation of clinical features occurring in the CDKL5 disorder and in Rett syndrome, reinforcing the concept that CDKL5 is an independent disorder with its own distinctive characteristics.

## Background

The CDKL5 disorder is a rare X-linked neurodevelopmental disorder caused by a disruption in the cyclin dependent kinase-like 5 (C*DKL5*) gene [1]. Mutations in this gene were originally identified in children with West syndrome, X-linked infantile spasms and principally in those with the early seizure onset form of Rett syndrome [1, 2]. Later research however demonstrated that less than one quarter of CDKL5 cases would actually meet the criteria for this atypical form of Rett syndrome [3]. Therefore, the CDKL5 disorder has been further defined as an independent disorder, characterized by early-onset intractable seizures, severely impaired gross motor skills and global developmental delay with sleep disturbances, abnormal muscle tone, bruxism and gastrointestinal issues also occurring commonly [3]. Less common features are hand stereotypies, laughing and screaming spells, breathing disturbances, peripheral vasomotor disturbances and a range of dysmorphic features. The latter include a broad/prominent forehead, large “deep-set” eyes, full lips, tapered fingers and anteverted nares [3].

Rett syndrome is a well-studied rare neurodevelopmental disorder caused by a mutation in the methyl CpG binding protein 2 (MECP2) gene [4]. Like the CDKL5 disorder, it is associated with severe disability and multiple comorbidities including scoliosis, breathing disturbances and sleep abnormalities [5–12]. A worldwide infrastructure which includes the Australian and International Rett syndrome (InterRett) databases [13, 14] and the US Natural History study [15] has enabled the clinical picture and variation in Rett syndrome to be understood.

Unlike Rett syndrome, very little is known about the full range of comorbidities occurring in the CDKL5 disorder with available literature mainly limited to small case series or studies [1–3, 16–32]. The establishment of an international database for the CDKL5 disorder [23], a data repository designed to build a large sample size and facilitate future genotype-phenotype relationships, has provided opportunity to examine these poorly researched clinical aspects. The current study has used this database to examine the prevalence of comorbidities of epilepsy, gastrointestinal problems including feeding difficulties, sleep and respiratory problems and scoliosis in individuals with the CDKL5 disorder, and their relationships with age and genotype. The prevalence and onset of comorbidities in the CDKL5 disorder were then compared with those occurring in Rett syndrome.

## Method

### Data sources

Data for the CDKL5 disorder were sourced from the International CDKL5 Disorder Database (ICDD) with supplementation of a small number of cases from InterRett, the International Rett Syndrome Database. The ICDD (http://cdkl5.childhealthresearch.org.au) was established in 2012 with the purpose of collecting information internationally on individuals with the CDKL5 disorder. A CDKL5 disorder specific questionnaire was formulated to capture comprehensively the clinical features and natural history of the disorder [23]. Individuals were included in this study if their *CDKL5* mutation was considered to be pathogenic or probably pathogenic and when at least one relevant section of the questionnaire had been completed. As the CDKL5 disorder was initially considered to be an atypical form of Rett syndrome, data on individuals with the CDKL5 disorder were initially included in InterRett, an international phenotype database that collects data worldwide on the clinical features and genetic characteristics of individuals with Rett syndrome [14]. For the majority of these cases, the data subsequently provided to ICDD was used for this study, but for a small proportion ICDD data was not available and thus we used InterRett data to allow inclusion of these cases in the study.

Data for Rett syndrome comorbidities was available from follow-up data collected up to 2011 in the population-based Australian Rett Syndrome Database (ARSD), a longitudinal database that collects information from families and clinicians [33]. Questionnaires are administered on recruitment and since 2000, additional follow-up questionnaires have also been sent to families every 2 to 3 years [34].

Ethics approval for this study was provided by the University of Western Australia Human Research Ethics Committee (RA/4/1/5024).

#### Covariates

The covariates included age group, gender, mutation type and for comparative purposes the type of disorder (CDKL5 disorder and Rett syndrome). Age at registration to ICDD was grouped into three categories (< 5 years, 5 to < 10 years, ≥ 10 years) consistent with the age distribution of the study sample, which comprised mainly of young children and few adults (9/167, 5.4 %). Due to the large number of different individual CDKL5 gene mutations and the few recurrent mutations, the mutation types were categorized as previously [23] according to their predicted structural and functional consequences [35]. Mutations were grouped as: (A) no functional protein, comprising any mutation that prevents function in the catalytic domain; (B) missense/in-frame mutations within catalytic domain, including any missense mutation within the protein kinase active region or in-frame mutation that results in loss of some kinase region (with consequent protein intact) due to deletion; (C) truncation occurring between aa172 and aa781, including any truncations such as nonsense or frameshift mutations that cause loss of c-terminal region whilst maintaining kinase activity; and (D) late truncation occurring after aa781 including truncations that maintains the kinase activity and a large portion of c-terminal region [35].

#### Outcomes

##### CDKL5

Age of onset and current seizure frequency (daily, weekly, monthly or less frequent than monthly) were used to characterise epilepsy. Parents were asked to report whether their child had ever and currently had constipation, reflux and air swallowing; the method of feeding (oral, enteral or a combination); ability to self-feed (independent, assisted or dependent) restricted to children aged 2 years or more, the occurrence/frequency of coughing during meals for those who fed orally; and the age at gastrostomy insertion. They were also requested to report on the presence of any sleep problems and specific problems of night waking and night laughing, and to complete the Sleep Disturbances Scale for Children (SDSC). The SDSC is a 26 item self-report scale, which allows parents to rate their children’s sleep behaviour using a 5-point Likert scale during the previous 6 months [36]. Information was sought on respiratory problems included the presence and frequency of breathing irregularities (hyperventilation, breath holding) such as those found in Rett syndrome [11]. Furthermore, we asked parents to report on episodes of respiratory illnesses including pneumonia and aspiration over their child’s life course, and also on their trajectory of hospitalisations by cause. The impacts of lower respiratory tract infections over 5-year intervals were also estimated by parents. Information was also requested on the presence of scoliosis, age of diagnosis and type of treatment (physical, bracing, spinal surgery).

##### Rett syndrome

Information was available on age of onset of epilepsy, and gastrostomy insertion; and the presence of sleep problems, gastrointestinal problems and irregular breathing patterns at various time-points in the life of the individual. The occurrence of lower respiratory tract infections over the life course prior to the initial follow-up questionnaire and during previous 12-month periods for subsequent follow-up questionnaires was also available. Recently published information was used for estimates of age of onset of scoliosis [37].

#### Statistical analyses

Descriptive statistics were used to characterise the comorbidities (epilepsy, gastrointestinal problems, feeding difficulties, sleep abnormalities, respiratory problems and scoliosis) in individuals with *CDKL5* disorder and Rett syndrome. Logistic regression (multivariate and univariate) was used to analyse the associations between age group, mutation type and the prevalence of various comorbidities. Multinomial logistic regression was used to investigate the relationship between the frequencies of coughing during meals and the independent variables. Odds ratios and beta coefficient values were examined to interpret the effect sizes. Sleep scores (SDSC) of individuals with CDKL5 disorder were compared with a normal population sample [36] using two-sample *t*-test with unequal variance. The incidence rate of hospitalisation (in 100 person-years) was determined using the number of hospitalisations over the total person-time of the hospitalised individuals.

The comparison with Rett syndrome was restricted to females for both disorders such that males with the CDKL5 disorder were excluded. Binary longitudinal data from the ARSD and the equivalent cross-sectional data from ICDD were examined using generalized linear model with generalized estimating equations. This accounted for clustering within individuals, to estimate the effect of the type of disorder on the likelihood of selected comorbidities after adjusting for age. Log link function, robust standard errors and exchangeable working correlation structures were used in the model for parameter estimation. For time to event analysis, the Kaplan-Meier method was used to estimate the failure function for the two disorders and the log-rank test was used to compare the two functions. Event time was defined as the age at onset of scoliosis, epilepsy or gastrostomy or the age of ascertainment if censored. STATA (Version 13, StataCorp, College Station, TX, USA) was used for statistical analysis.

## Results

As of June 2015, there were 167 confirmed cases with a pathogenic *CDKL5* mutation registered either with ICDD (*n* = 151) or InterRett (*n* = 16). Age at registration ranged from 3.9 months to 29.1 years (median age 5.9 years). The majority (85.6 %, *n* = 143) were females (median age 5.9 years, range 3.9 months to 29.1 years) and 14.4 % (*n* = 24) were males (median age 5.8 years, range 10.5 months to 24.5 years). Sufficient mutation details were available for 164 cases to enable classification into mutation groups based on the structure and function of the *CDKL5* gene. Case distribution, according to gender, age group, mutation group and comorbidities is shown in Table 1, and the distribution of comorbidities by mutation group is shown in Table 2.

**Table 1:** Frequency distribution of age, mutation type and prevalence of comorbidities by gender

	Female (*n* = 143)	Male (*n* = 24)	Total (*n* = 167)
	n/N (%)	n/N (%)	n/N (%)
Age group			
0 < 5 years	58/143 (40.6)	12/24 (50.0)	70/167 (41.9)
5 < 10 years	49/143 (34.3)	6/24 (25.0)	55/167 (32.9)
10 years or older	36/143 (25.2)	6/24 (25.0)	42/167 (25.2)
Mutation group			
No functional protein	41/141 (29.1)	10/23 (43.5)	51/164 (31.1)
Missense/in-frame mutation within catalytic domain	39/141 (27.7)	6/23 (26.1)	45/164 (27.4)
Truncation between aa172 and aa781	37/141 (26.2)	1/23 (4.4)	38/164 (23.2)
Truncation after aa781	15/141 (10.6)	2/23 (8.7)	17/164 (10.4)
Mutation not grouped	9/141 (6.4)	4/23 (17.4)	13/164 (7.9)
Epilepsy ever	128/130 (98.5)	17/18 (94.4)	145/148 (98.0)
GI health			
Any GI problem ever	106/123 (86.2)	16/18 (88.9)	122/141 (86.5)
Constipation ever	85/123 (69.1)	15/18 (83.3)	100/141 (70.9)
Reflux ever	72/111 (64.9)	10/17 (58.8)	82/128 (64.1)
Air swallowing ever	32/116 (27.6)	4/17 (23.5)	36/133 (27.1)
Gastrostomy ever	31/127 (24.4)	11/19 (57.9)	42/146 (28.8)
Sleep^a^			
Any sleep problem ever	106/124 (85.5)	16/17 (94.1)	122/141 (86.5)
Night waking	63/106 (59.4)	9/17 (52.9)	72/123 (58.5)
Diurnal problems^b^	51/108 (47.2)	8/18 (44.4)	59/126 (46.8)
Teeth grinding	41/108 (38.0)	9/18 (50.0)	50/126 (39.7)
Night screaming	25/109 (22.9)	4/18 (22.2)	29/127 (22.8)
Night laughing	28/109 (25.7)	6/18 (33.3)	34/127 (26.8)
Repetitive actions while falling asleep	29/107 (27.1)	4/18 (22.2)	33/125 (26.4)
Respiratory			
Any breathing irregularities	33/110 (30.0)	8/16 (50.0)	41/126 (32.5)
Hyperventilation	15/109 (13.8)	2/16 (12.5)	17/125 (13.6)
Breath-holding	26/109 (23.9)	7/16 (43.8)	33/125 (26.4)
Pneumonia ever	18/109 (16.5)	9/17 (52.9)	27/126 (21.4)
Aspiration	19/107 (17.8)	9/17 (52.9)	28/124 (22.6)
Scoliosis	23/122 (18.9)	5/16 (31.3)	28/138 (20.3)

^a^Individuals with frequent sleep problems (*1–2 times per week, 3–5 times per week and daily*) only

^b^Diurnal problems: daytime napping

**Table 2:** Prevalence of comorbidities by mutation group in 164 individuals with the *CDKL5* disorder

	No functional protein (*n* = 51)	Missense/in-frame mutation within catalytic domain (*n* = 45)	Truncation between aa172 and aa781 (*n* = 38)	Truncation after aa781 (*n* = 17)
	n/N (%)	n/N (%)	n/N (%)	n/N (%)
Epilepsy ever	47/48 (97.6)	39/40 (96.8)	32/32 (100.0)	16/16 (100.0)
GI health				
Any GI problem	30/43 (69.8)	28/38 (73.7)	18/30 (60.0)	9/14 (64.3)
Constipation	20/43 (46.5)	23/40 (57.5)	20/31 (64.5)	6/15 (40.0)
Reflux ever	16/40 (40.0)	17/34 (50.0)	11/29 (37.9)	3/13 (23.1)
Air swallowing	2/41 (4.9)	10/38 (26.3)	7/30 (23.3)	4/15 (26.7)
Gastrostomy ever	14/45 (31.1)	14/41 (34.2)	8/32 (25.0)	3/16 (18.8)
Sleep^a^				
Any sleep problem ever	41/45 (91.1)	35/40 (87.5)	24/30 (80.0)	14/16 (87.5)
Persistent night waking	26/39 (66.7)	21/33 (63.6)	14/28 (50.0)	6/12 (50.0)
Diurnal problems^b^	18/41 (43.9)	19/34 (55.9)	13/28 (46.4)	6/12 (50.0)
Teeth grinding	15/40 (37.5)	13/34 (38.2)	11/28 (39.3)	4/13 (30.8)
Night screaming	11/40 (27.5)	12/35 (34.3)	2/28 (7.1)	2/13 (15.4)
Night laughing	12/40 (30.0)	12/35 (34.3)	6/28 (21.4)	1/13 (7.7)
Repetitive actions while falling asleep	11/40 (27.5)	8/33 (24.2)	6/28 (21.4)	4/13 (30.8)
Respiratory				
Any breathing irregularities	12/41 (29.3)	14/35 (40.0)	8/30 (26.7)	1/9 (11.1)
Hyperventilation	3/44 (6.8)	3/34 (17.7)	5/30 (16.7)	1/9 (11.1)
Breath-holding	11/41 (26.8)	11/33 (33.3)	6/30 (20.0)	1/10 (10.0)
Pneumonia ever	10/42 (23.8)	5/33 (15.2)	6/28 (21.4)	3/12 (25.0)
Aspiration ever	12/41 (29.3)	6/33 (18.2)	6/27 (22.2)	1/12 (8.3)
Scoliosis	9/44 (20.5)	9/40 (22.5)	7/30 (23.3)	2/15 (13.3)

^a^indicates that only individuals with frequent sleep problems (*1–2 times per week, 3–5 times per week and daily rather than monthly or less frequently*) were classified as having the sleep problem

^b^Diurnal problems: daytime napping

### Epilepsy in the CDKL5 disorder

Families of all (98.0 %, 145/148) but two females and one male reported that their child had experienced one or more episodes of seizures. The median age of epilepsy onset was 6 weeks (range 1 day to 1.5 years). Information on current seizure frequency was available for 137/145. Ninety-five individuals (69.3 %, 95/137) were experiencing seizures daily with their average frequency of daily seizures ranging from one to 21 seizures. Of those who provided information on the number of daily seizures (*n* = 82), the majority (56/82) were experiencing fewer than five seizures a day, 18/82 between five and ten seizures, and 8/82 more than 10 seizures every day. Otherwise, almost 13.9 % (19/137) were having seizures approximately weekly, 9.5 % (13/137) monthly and one individual (0.7 %) only occasionally experienced seizures. Nine individuals (6.6 %) had had no seizures for more than a year.

### Gastro-intestinal health in the CDKL5 disorder

The majority (86.5 %, 122/141) of the individuals were reported to have experienced at least one gastrointestinal (GI) problem including constipation (70.9 %), reflux (64.1 %) and air swallowing (27.1 %) (Table 1). Children aged 10 years or older had 3.5 times the odds of currently experiencing a GI problem compared to children under 5 years (odds ratio (OR) 3.5, 95 % confidence interval (CI) 1.22, 10.08) after adjusting for mutation (Table 3). Compared to the no functional protein group, individuals with a missense/in-frame mutation within catalytic domain and truncation after aa172 had higher odds of air swallowing (Table 3). The odds of experiencing constipation, reflux or air swallowing also increased with age, especially in those over 10 years (Table 3). The odds of experiencing constipation (OR 0.90, 95 % CI 0.28, 2.90) or reflux (OR 1.01, 95 % CI 0.31, 3.34) were similar for both males and females.

**Table 3:** Multivariate logistic regression analysis of current gastrointestinal problems by age, gender and mutation group (*n* = 164)

	Any GI problem			Constipation			Reflux			Air swallowing		
	(*n* = 94)			(*n* = 73)			(*n* = 51)			(*n* = 25)		
	*n* (%)	OR (95 % CI)	*p* value	*n* (%)	OR (95 % CI)	*p* value	*n* (%)	OR (95 % CI)	*p* value	*n* (%)	OR (95 % CI)	*p* value
	*N* = 125			*N* = 129			*N* = 116			*N* = 110		
Age group												
0 to <5 years	50 (40.0)	baseline	–	52 (40.3)	baseline	–	48 (41.4)	baseline	–	44 (40.0)	baseline	–
5 to < 10 years	42 (33.6)	2.03 (0.83,4.94)	0.12	42 (32.6)	2.12 (0.90,5.00)	0.08	36 (31.0)	1.07 (0.42,2.72)	0.88	37 (33.6)	1.35 (0.37,4.86)	0.65
10 years or older	33 (26.4)	3.5 (1.22,10.08)	0.02	35 (27.1)	2.78 (1.14,6.74)	0.02	32 (27.6)	2.24 (0.88,5.71)	0.09	29 (26.4)	7.48 (1.96,28.53)	0.003
Gender												
Female	112 (89.6)	baseline	–	115 (89.2)	baseline	–	102 (87.9)	baseline	–	110 (100)	baseline	–
Male	13 (10.4)	0.92 (0.25,3.44)	0.90	14 (10.9)	0.90 (0.28,2.90)	0.85	14 (12.1)	1.01 (0.31,3.34)	0.99	0	–	–
Mutation group												
No functional protein	43 (34.4)	baseline	–	43 (33.3)	baseline	–	40 (34.5)	baseline	–	34 (30.9)	baseline	–
Missense/in-frame mutation within catalytic domain	38 (30.4)	1.27 (0.46,3.50)	0.64	40 (31.0)	1.62 (0.65,4.03)	0.30	34 (29.3)	1.73 (0.66,4.51)	0.27	34 (30.9)	12.14 (2.08,70.68)	0.01
Truncation between aa172 and aa781	30 (24.0)	0.67 (0.24,1.89)	0.45	31 (24.0)	2.17 (0.80,5.91)	0.13	29 (25.0)	1.01 (0.36,2.82)	0.98	29 (26.4)	8.03 (1.35,48.00)	0.02
Truncation after aa781	14 (11.2)	0.81 (0.21,3.07)	0.76	15 (11.6)	0.74 (0.21,2.58)	0.64	13 (11.2)	0.49 (0.11,2.13)	0.34	13 (11.8)	14.21 (1.83,110.22)	0.01

*OR* odds ratio - the relative odds of having the outcome in the category of interest compared to the odds of having the outcome in the baseline category; *CI* confidence interval

### Feeding difficulties and methods of feeding in the CDKL5 disorder

Nearly four-fifths of the affected individuals (79.3 %, 107/135) were reported to eat and drink orally. Of these, 12 children had had a feeding tube inserted for other purposes such as venting gas and administration of nutritional supplements or medications. Twenty-eight individuals (20.7 %) were exclusively enterally fed (gastrostomy tube or nasogastric tube feeding). Use of nasogastric tube feeding was uncommon (2.2 %, 3/135).

Feeding difficulties were reported for over a half (50.9 %, 48/95) of the orally fed individuals with 60 % (45/75) of the children over 2 years of age completely dependent on their families for eating and drinking. A much smaller number (5.3 %, 4/75) of children were able to eat/drink independently; some (16.0 %, 12/75) could finger feed with assistance or were assisted with spoon-feeding (18.7 %, 14/75). Almost three-quarters (73.4 %, 69/94) of the parents reported at least occasional (>1/week) coughing during oral feeding. There were some evidence to suggest that the relative risk of coughing (sometimes or often vs never) during feeding was greatest in those under 5 years (Table 4). Relationships between coughing, gender and genotype are also shown in Table 4.

**Table 4:** Multinomial logistic analysis of frequency of coughing by age group, gender and mutation group

	Do not occur	Sometimes occur			Often occur		
	(*n* = 25)	(*n* = 47)			(*n* = 21)		
	*n* (%)	*n* (%)	RRR (95 % CI)	*p* value	*n* (%)	RRR (95 % CI)	*p* value
Age group							
0 to <5 years	7 (28.0)	24 (54.6)	–	–	13 (61.9)	–	–
5 to <10 years	10 (40.0)	13 (46.4)	0.26 (0.07,1.01)	0.05	5 (23.8)	0.13 (0.02,0.73)	0.02
10 years or older	8 (32.0)	11 (22.9)	0.25 (0.06,0.98)	0.05	3 (14.3)	0.16 (0.03,0.91)	0.04
Gender							
Female	23 (92.0)	44 (91.7)	–	–	19 (90.5)	–	
Male	2 (8.0)	4 (8.3)	1.83 (0.17,20.20)	0.62	2 (9.5)	0.80 (0.04,16.47)	0.88
Mutation group							
No functional protein	8 (32.0)	15 (31.9)	–	–	–	–	–
Missense/in-frame mutation within catalytic domain	6 (24.0)	12 (25.5)	1.05 (0.26,4.32)	0.94	6 (28.6)	1.32 (0.25,7.13)	0.74
Truncation between aa172 and aa781	6 (24.0)	12 (25.5)	1.30 (0.31,5.48)	0.72	4 (19.1)	1.10 (0.18,6.67)	0.92
Truncation after aa781	2 (8.0)	5 (10.6)	1.23 (0.19,8.05)	0.83	3 (14.3)	2.36 (0.26,21.16)	0.44

*RRR* relative risk ratio; *CI* confidence interval

Frequency of coughing grouped as the following: - do not occur (never); sometimes (less than 1/week and 1–2 times per week); often occur (daily and more than 1/day)

### Gastrostomy/jejunostomy in the CDKL5 disorder

Over a quarter (28.8 %, 42/146) of individuals had had a gastrostomy or jejunostomy tube inserted due to various and sometimes multiple reasons including feeding difficulties (80.0 %), poor weight gain (60.0 %), difficulty with administration of medications (62.9 %) and poor general health and wellbeing (51.4 %).

**Fig. 1:**
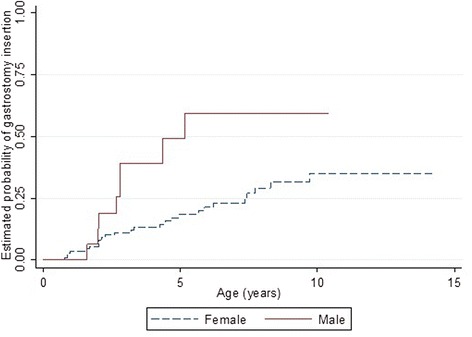
Estimated time-to-event functions of gastrostomy insertion for males and females with the CDKL5 disorder

Time to event analysis showed that males were more likely to undergo gastrostomy than females (log rank test *p* = 0.013) with a quarter of males having gastrostomy insertion by age 2.6 (95 % CI 1.60, 4.34) compared to 7.4 years (95 % CI 4.64,-) for females (Fig. 1). No particular differences were observed among the mutation groups (log rank test *p* = 0.50).

### Sleep in the CDKL5 disorder

The majority (86.5 %, 122/141) of individuals were reported to have experienced a sleep problem during their lifetime with current night waking occurring in 58.5 % (72/123) (Table 1). The odds of sleep problems overall were higher in the 5 to 10 year age group compared with those younger than 5 years (OR 13.09, 95 % CI 1.57, 109.02), while the odds of night waking was lower in those with a truncation after aa172 than the no functional protein group (Table 5).

**Table 5:** Multivariate logistic analyses of any and specific current sleep problems by age, gender and mutation group

	Any sleep problem			Night waking			Night screaming			Night laughing		
	(*n* = 122)			(*n* = 72)			(*n* = 29)			(*n* = 34)		
	*n* (%)	OR (95 % CI)	*p* value	*n* (%)	OR (95 % CI)	*p* value	*n* (%)	OR (95 % CI)	*p* value	*n* (%)	OR (95 % CI)	*p* value
	*N* = 131			*N* = 112			*N* = 116			*N* = 125		
Age group												
0 to <5 years	53 (40.5)	baseline	–	48 (42.9)	baseline	–	47 (40.5)	baseline	–	53 (42.4)	baseline	–
5 to < 10 years	42 (32.1)	13.09 (1.57,109.02)	0.02	36(32.1)	9.16 (2.91,28.86)	<0.001	38 (32.8)	1.01 (0.34,3.02)	0.98	40 (32.0)	0.96 (0.32,2.92)	0.95
10 years or older	36 (27.5)	1.48 (0.45,4.84)	0.52	28 (25.0)	1.80 (0.67,4.85)	0.24	31 (26.7)	1.22 (0.41,3.67)	0.72	32 (25.6)	3.38 (1.20,9.53)	0.02
Gender												
Female	116 (88.6)	baseline	–	98 (87.5)	baseline	–	101 (87.1)	baseline	–	107 (85.6)	baseline	–
Male	15 (11.5)	2.37 (0.27,20.53)	0.43	14 (12.5)	0.98 (0.27,3.53)	0.98	15 (12.9)	0.63 (0.16,2.53)	0.52	18 (14.4)	1.86 (0.38,4.05)	0.33
Mutation group												
No functional protein	45 (34.4)	baseline	–	39 (34.8)	baseline	–	40 (34.5)	baseline	–	40 (34.5)	baseline	–
Missense/in-frame mutation within catalytic domain	40 (30.5)	0.59 (0.14,2.49)	0.48	33 (29.5)	0.74 (0.26,2.12)	0.58	35 (30.2)	1.37 (0.50,3.78)	0.54	35 (30.2)	1.67 (0.58,4.78)	0.34
Truncation between aa172 and aa781	30 (22.9)	0.32 (0.08,1.33)	0.12	28 (25.0)	0.32 (0.10,0.99)	0.05	28 (24.1)	0.19 (0.04,0.98)	0.05	28 (24.1)	0.87 (0.26,2.89)	0.82
Truncation after aa781	16 (12.2)	0.41 (0.06,2.78)	0.37	12 (10.7)	0.24 (0.05,1.08)	0.06	13 (11.2)	0.48 (0.09,2.63)	0.40	13 (11.2)	0.22 (0.02,1.98)	0.18

*OR* odds ratio - the relative odds of having the outcome in the category of interest compared to the odds of having the outcome in the baseline category; *CI* confidence interval

**Fig. 2:**
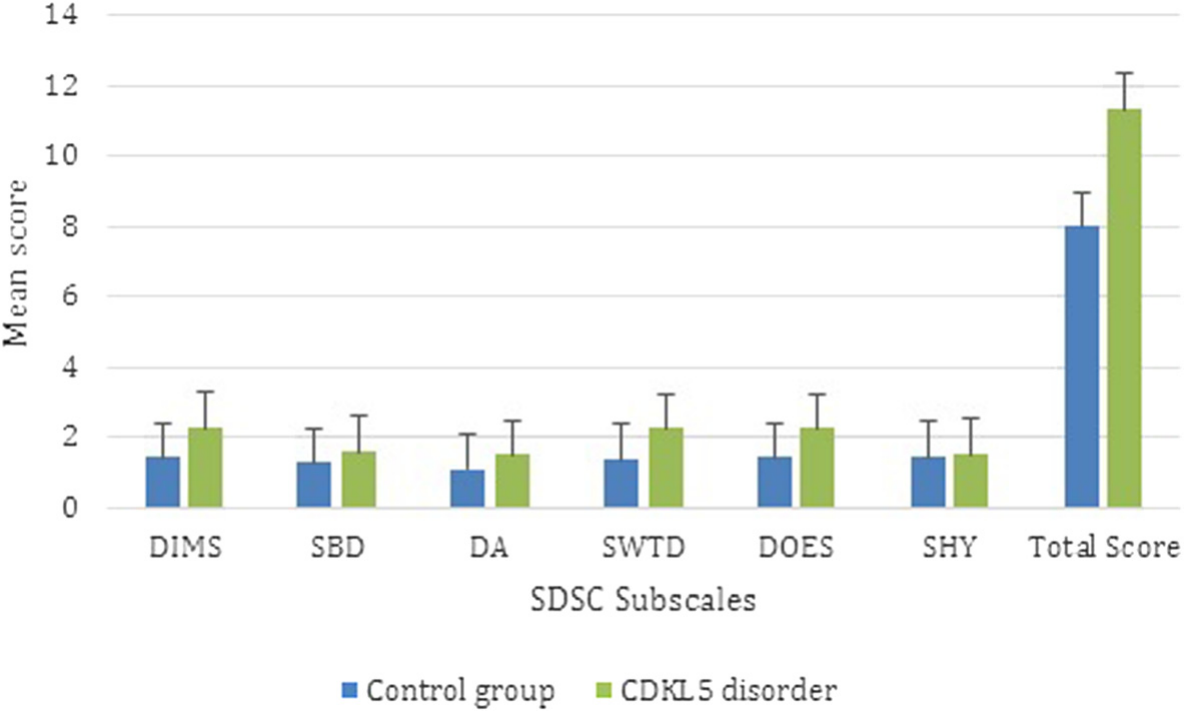
Mean subscale scores of the SDSC in *CDKL5* positive individuals (*n* = 124) and published controls (*n* = 1157). Figure legend: DIMS, disorders of initiating and maintaining sleep; SBD, sleep breathing disorders; DA, disorders of arousal; SWTD, sleep-wake transition disorders; DOES, disorders of excessive somnolence; SHY, sleep hyperhidrosis. Subscale scores ranges from 1 to 5 with highest score possible is 5 with higher scores indicating more problematic sleep. The group data describing the healthy controls were derived from Bruni et al. [36]

The mean overall sleep disturbance score in those with the CDKL5 disorder was higher than that in published control subjects (two-sample *t*-test with unequal variance *p* < 0.001) [38] (Fig. 2). The majority of subscale scores including disorders of initiating and maintaining sleep, sleep breathing disorders, disorders of arousal, sleep-wake transition disorders and disorders of excessive somnolence were also higher (*p* < 0.001) (Fig. 2).

### Breathing abnormalities in the CDKL5 disorder

Almost one-third (32.5 %, 41/126) were reported to have breathing irregularities at the time of registration. These included hyperventilation (13.6 %, 17/125) and breath holding (26.4 %, 33/125) (Table 1). A history of pneumonia was reported for 21.4 % (27/126) and aspiration for 22.6 % (28/124) (Table 1). The odds of having an autonomic-type breathing irregularity was higher in older individuals than children younger than 5 years of age but the level of evidence was low (Table 6). Males were more vulnerable than females to experiencing apparent autonomic dysfunction such as breath holding (OR 4.71, 95 % CI 1.30, 16.99), as well as infections such as pneumonia (OR 7.51, 95 % CI 2.16, 26.09).

**Table 6:** Multivariate logistic regression analysis of respiratory problems by gender and mutation type

	Any autonomic dysfunction			Breath-holding			Hyperventilation			Respiratory problem^ab^			Pneumonia ever^b^		
	(*n* = 41)			(*n* = 33)			(*n* = 17)			(*n* = 28)			(*n* = 27)		
	n (%)	OR (95 % CI)	*p* value	n (%)	OR (95 % CI)	*p* value	n (%)	OR (95 % CI)	*p* value	n (%)	OR (95 % CI)	*p* value	n (%)	OR (95 % CI)	*p* value
	*N* = 115			*N* = 114			*N* = 117			*N* = 111			*N* = 115		
Age group															
0 to <5 years	47 (40.9)	baseline	–	46 (40.4)	baseline	–	49 (41.9)	baseline	–	44(39.6)	–	–	47 (40.9)	–	–
5 to 10 years	39 (33.9)	1.83 (0.68,4.95)	0.24	40 (35.1)	2.15 (0.74,6.19)	0.16	38 (32.5)	1.92 (0.49,7.59)	0.35	37(33.3)	–	–	37 (32.2)	–	–
10 years or older	29 (25.2)	2.18 (0.76,6.25)	0.15	28 (24.6)	1.72 (0.54,5.45)	0.36	30 (25.6)	2.58 (0.61,10.83)	0.20	30(27.0)	–	–	31 (27.0)	–	–
Gender															
Female	102 (88.7)	baseline	–	101 (88.6)	baseline	–	104 (88.9)	baseline	–	97 (87.4)	baseline	–	101 (87.8)	baseline	–
Male	13 (11.3)	3.65 (1.02,13.04)	0.05	13 (11.4)	4.71 (1.30,16.99)	0.02	13 (11.1)	0.70 (0.08,6.23)	0.75	14 (12.6)	5.84 (1.67,20.47)	0.01	14 (12.2)	7.51 (2.16,26.09)	0.002
Mutation group															
No functional protein	41 (35.7)	baseline	–	41 (36.0)	baseline	–	44 (37.6)	baseline	–	40 (36.0)	baseline	–	42 (36.5)	baseline	–
Missense/in-frame mutation within catalytic domain	35 (30.4)	1.94 (0.69,5.48)	0.21	33 (29.0)	1.56 (0.52,4.68)	0.43	34 (29.1)	3.02 (0.67,13.65)	0.15	33 (29.7)	1.17 (0.34,3.99)	0.80	33 (28.7)	0.77 (0.21,2.84)	0.70
Truncation between aa172 and aa781	30 (26.1)	1.12 (0.36,3.46)	0.85	30 (26.3)	0.85 (0.25,2.88)	0.80	30 (25.6)	2.69 (0.55,13.03)	0.22	26 (23.4)	1.13 (0.30,4.30)	0.86	28 (24.4)	1.42 (0.40,5.03)	0.58
Truncation after aa781	9 (7.8)	0.30 (0.03,2.76)	0.30	10 (8.8)	0.29 (0.03,2.72)	0.28	9 (7.7)	1.52 (0.13,17.50)	0.74	12 (10.8)	0.65 (0.11,3.84)	0.63	12 (10.4)	1.18 (0.23,6.08)	0.85

*OR* odds ratio - the relative odds of having the outcome in the category of interest compared to the odds of having the outcome in the baseline category; *CI* confidence interval

^a^Respiratory problems that required hospital admission or doctor consultation

^b^Prevalence of respiratory problems and pneumonia were not analysed by age group as the question asked about past history not current occurrence

Families were asked to estimate the adverse impacts (occasional or constant) of other acute illnesses in 5-year intervals over their child’s lifetime (Table 7). During the first 5 years of life, half (50.0 %, 62/124) of the families reported that their child experienced problems due to ear infections and over a third (37.1 % (46/124)) reported lower respiratory tract infections (LRTI) such as pneumonia, bronchitis and bronchiolitis (Table 7). All acute illnesses including lower respiratory tract infections and urinary tract infection became less problematic after the age of 15 years (Table 7). Seizures, respiratory infections, and other acute illnesses accounted for a total of 531 hospitalisations.

**Table 7:** Frequency distribution of various acute illnesses being sometimes or constantly problematic during 5-year epochs

	<5 years	5 - < 10 years	10- <15 years	15 - < 20 years	Over 20 years
	n/N (%)	n/N (%)	n/N (%)	n/N (%)	n/N (%)
Lower Respiratory Tract Infection^a^	46/124 (37.1)	12/58 (20.7)	18/23 (21.7)	1/9 (11.1)	1/8 (12.5)
Asthma	10/113 (8.1)	8/58 (13.8)	3/22 (13.6)	0/9 (0)	0/7 (0)
Urinary Tract Infection	21/123 (17.1)	13/56 (23.2)	6/23 (26.1)	1/9 (11.1)	0/7 (0)
Ear infection	62/124 (50.0)	14/56 (25.0)	2/22 (9.1)	1/8 (12.5)	0/7 (0)
Tonsillitis	10/123 (8.1)	7/58 (12.1)	2/23 (8.7)	0/9 (0)	0/7 (0)

The denominators of some variables are lower for older age groups due to children being younger at ascertainment and due to some missing data (some parents have not responded)

^a^Lower respiratory infection is comprised of infections including pneumonia, bronchitis and bronchiolitis

Seizure-related hospitalisations accounted for nearly two-thirds (63.5 %, 337/531) of these hospitalisations over 706.5 person-years with an incidence of 47.4 admissions per 100 person-years. For the children of the 69/91 families with seizure-related admissions who provided sufficient detail on these, the mean number of days in hospital due to seizure-related events was 27.4 (median 19 days, range 1 day to 4.9 months). Respiratory problems including aspiration, pneumonia, bronchitis and bronchiolitis accounted for 62 hospitalisations (11.7 %). The incidence of respiratory-related hospitalisations was 8.2 admissions per 100 person-years. Thirty-seven (29.1 %, 37/127) individuals had had at least one hospitalisation related to respiratory problems over their lifetime.

### Scoliosis in the CDKL5 disorder

**Fig. 3:**
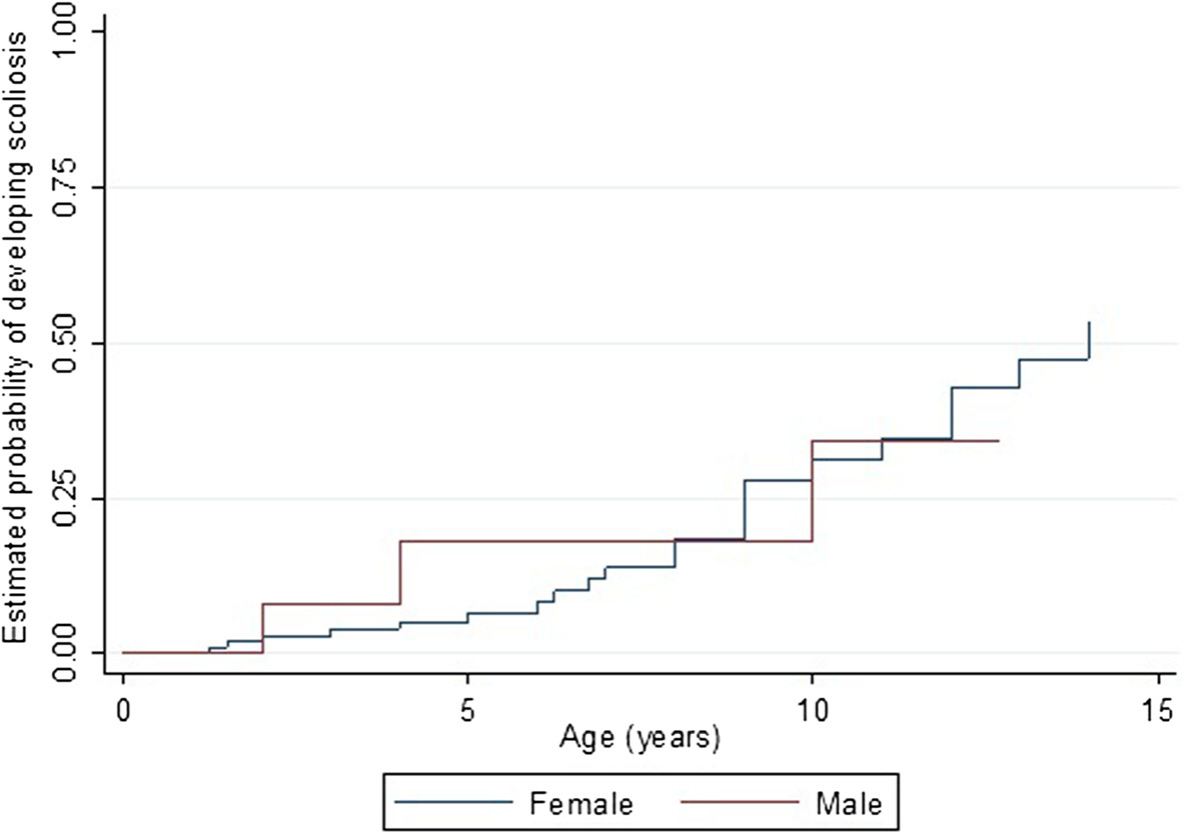
Estimated time-to-event functions of developing scoliosis for males and females with the CDKL5 disorder

The risk of developing scoliosis increased with age with a 68.5 % (95 % CI 0.53, 0.80) likelihood of developing scoliosis by age 10 years (Fig. 3). Physical treatments for scoliosis had been undertaken for 39.3 % (11/28) of the individuals and mainly included bracing treatment (8/11). Spinal surgery had been performed in 3 out of the 28 individuals (10.7 %).

### Comparison with Rett syndrome

Follow-up data at one or more collection points were available in the ARSD for 321 females with a pathogenic *MECP2* mutation. The median and range of ages of the affected individuals for each of the follow up questionnaires in the Australian Rett syndrome study were 13.0 years (2.0–24.6), 13.8 years (2.8–27.3), 15.0 years (2.3–29.1), 15.8 years (2.6–30.8), 17.9 years (3.0–34.2) and 17.8 years (2.6–35.5) for the questionnaire years 2000, 2002, 2004, 2006, 2009 and 2011 respectively.

**Fig. 4:**
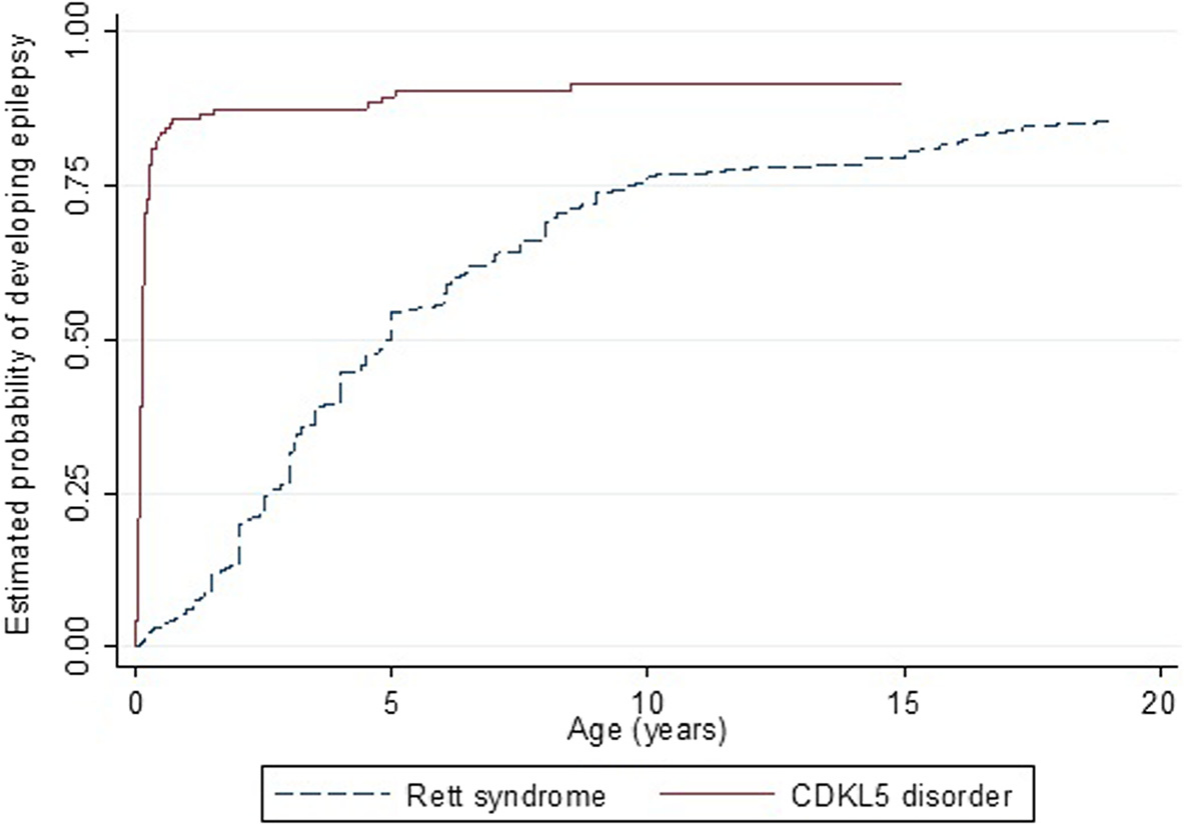
Estimated time-to-event functions of developing seizures for females with CDKL5 and Rett syndrome

The median age of epilepsy onset was much earlier in females with the CDKL5 disorder (6.0 weeks) than Rett syndrome (4.9 years) (Fig. 4) and the risk of developing seizures was much greater in CDKL5 affected females (log rank test *p* <0.001) during the observation period.

**Fig. 5:**
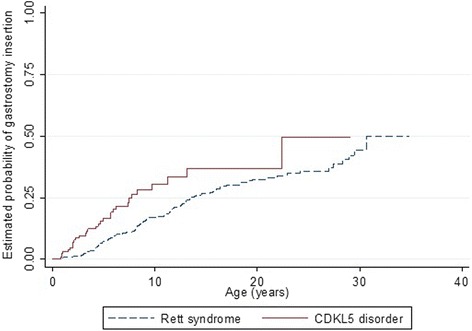
Estimated time-to-event functions of gastrostomy insertion for females with *CDKL5* disorder and Rett syndrome

**Fig. 6:**
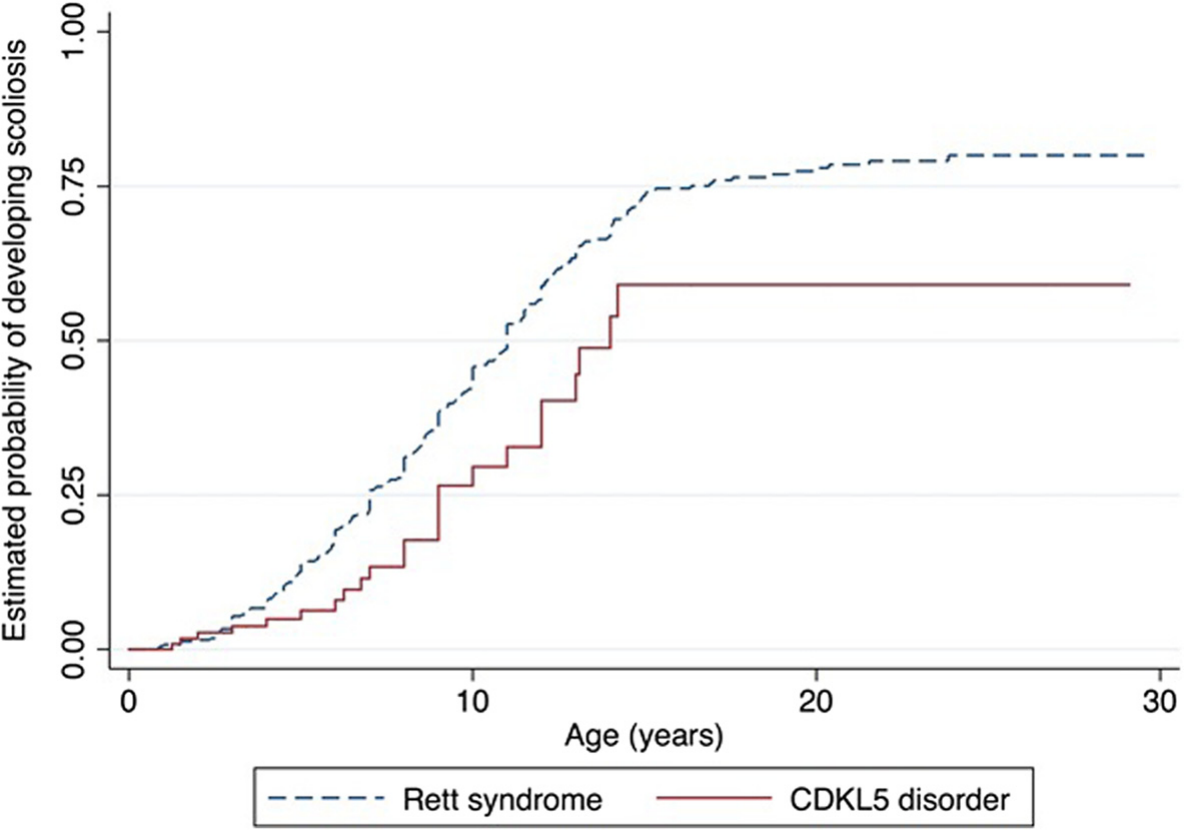
Estimated time-to-event functions of developing scoliosis for *CDKL5* and Rett syndrome females

Sleep problems were highly prevalent in both disorders but while night waking was more prevalent in the CDKL5 disorder than Rett syndrome, night laughing was less prevalent as were gastrointestinal problems (except for reflux), and autonomic-type breathing problems (Table 8). However, the incidence of gastrostomy insertion was higher in the CDKL5 disorder affected females (3.2 insertions per 100 person-years) than in Rett syndrome (1.9 insertions per 100 person-years). Females with CDKL5 disorder were more likely to have undergone gastrostomy at an earlier age (log rank test (log rank test *p* = <0.001) (Fig. 5). Compared with Rett syndrome, the relative risks of having breathing irregularities such as hyperventilation (RR 0.26, 95 % CI 0.16, 0.41) and breath holding (RR 0.40, 95 % CI 0.28, 0.57) were lower in CDKL5 disorder affected females. For respiratory infections, females with the CDKL5 disorder had a slightly greater risk of having experienced pneumonia when compared to Rett syndrome (RR 1.24, 95 % CI 0.62, 2.51). However, those with Rett syndrome were more likely to develop scoliosis than those with the CDKL5 disorder (log rank test *p* = 0.002). The median age of scoliosis onset for females with the CDKL5 disorder (14 years, 95 % CI 12, -) was much later than in Rett syndrome (11 years, 95 % CI 10, 11.6) [39] (Fig. 6).

**Table 8:** Comparison of various comorbidities in females with CDKL5 disorder (*n* = 143) and Rett syndrome (*n* = 321)

Comorbidity type	Rett syndrome^a^	*CDKL5* disorder	RR (95 % CI)	*p* value
	*n* (%)	*n* (%)		
GI health				
Any GI Problem	936/1181 (79.3)	82/120 (68.3)	0.85 (0.75–0.98)	0.02
Constipation	871/1181 (73.8)	65/123 (52.9)	0.72 (0.60–0.87)	<0.001
Reflux	214/793 (27.0)	44/110 (40.0)	1.41 (1.03–1.92)	0.03
Respiratory				
Hyperventilation	639/1175 (54.4)	15/109 (13.8)	0.26 (0.16–0.41)	<0.001
Breath-holding	724/1176 (61.6)	26/109 (23.9)	0.40 (0.28–0.57)	<0.001
Sleep				
Night waking^b^	422/591 (71.4)	96/110 (87.3)	1.18 (1.1–1.31)	0.03
Night screaming	483/1139 (42.4)	60/118 (50.9)	1.1 (0.86–1.32)	0.57
Night laughing	774/1139 (68.0)	70/122 (57.4)	0.76 (0.64–0.90)	0.001

Each individual in the AussieRett database could have contributed from one to six observations according to their participation in the initial and follow-up questionnaires, which accounts for the large number of total observation (Rett syndrome)

*RR* risk ratio; *CI* confidence interval

^a^Average prevalence for the 6 time points

^b^Data for females with night waking (Rett syndrome) was only available from 2009 and 2011 questionnaires

## Discussion

This large-sample study using a CDKL5 disorder specific database is the first to examine the comorbidities of the CDKL5 disorder in depth and to compare their occurrence with that of Rett syndrome. Epilepsy, GI problems and sleep abnormalities were common in the CDKL5 disorder whilst scoliosis and respiratory irregularities were less prevalent than in Rett syndrome. The likelihood of experiencing epilepsy, GI problems, respiratory problems, and scoliosis increased with age and males were more vulnerable to respiratory and sleep problems than females. There was no clear relationship between mutation group and prevalence of comorbidities. However, mutations associated with late truncations after aa781 seemed to play some protective role against GI problems, autonomic breathing irregularities and sleep problems. It was also observed that there were differences in clinical features occurring in CDKL5 disorder and Rett syndrome, reinforcing the notion that CDKL5 is an independent disorder with its own distinctive characteristics.

Consistent with previous studies, the early onset of epilepsy (median age of onset 6 weeks) was a key feature of CDKL5 disorder [18, 20]. Over three quarters were experiencing at least one seizure daily. This highlights the medical challenges in caring for children with the CDKL5 disorder. As we had found previously in a smaller study [3], GI disorders including constipation and reflux were very common. For the first time we have provided information about feeding difficulties and the current method of feeding (oral or enteral feeding) in the CDKL5 disorder. In this study, only two thirds were still being fed orally and of these three-fifths were completely dependent on their families for eating and drinking. This likely relates to the marked motor impairments associated with this disorder [3]. Further, over half of those who were orally fed experienced feeding difficulties including dysphagia (abnormal swallowing) and related symptoms such as coughing. As demonstrated in the current study, one third had undergone a gastrostomy, many at a young age where the purpose could have been associated with assisting with feeding and/or epilepsy management [38]. Gastrostomy insertions have been associated with improved quality of life of both the caregivers and child by reducing the stress associated with feeding and also minimizing the dysphagia symptoms such as coughing during feeding [39, 40].

Our large sample size has allowed us to characterize sleep difficulties in the CDKL5 disorder in much greater depth than was possible in previous small case series [24, 27]. Night waking was the most persistently occurring sleep problem, experienced by more than half of the individuals in our study. In a French clinical study, 11/20 of affected females experienced sleep problems such as night waking with screaming spells [19]. Similar findings were demonstrated by Hagebeuk [24] and Pini et al. [27] in two other case studies (*n* = 4 and *n* = 10) where all subjects were observed to have experienced some sleep abnormalities including disorders of initiation and maintaining of sleep, sleep breathing disorders and daytime somnolence. In our study, the Sleep Disturbance Scale for Children was used to assess the various aspects of sleep disturbances in a large sample with *CDKL5* disorder for the first time. When compared to normal healthy children [36], the frequency of overall sleep disturbances (except for night sweating) was higher in individuals with the CDKL5 disorder. In particular, problems related to maintaining and initiating sleep such as night waking and sleep–wake disorders difficulties including startling while falling asleep were frequently reported in this study. Similar to other neurodevelopmental disorders such as Autism Spectrum Disorder and Angelman syndrome, genetic abnormalities that alter the neuronal pathways might be a contributing factor to these sleep problems [41–44]. Even though these mechanisms are unclear, other factors that might be associated with these sleep disturbances include abnormalities in the circadian rhythm, altered production of neurotransmitters such as serotonin, periodic limb movements, and presence of other comorbidities such as uncontrollable seizures, scoliosis and gastro-oesophageal reflux disease [42, 45, 46]. Sleep problems have significant negative impact on the quality of life of child and parents. Levels of parental stress have been reported to be high among families of children with neurodevelopmental disorders [47]. Perhaps in relation to poor child sleep, parents of these children are likely also to have poorer sleep, which could contribute to the increased likelihood of depression and anxiety compared with parents who do not have a child with a neurodevelopmental disorder [45, 48].

Autonomic breathing irregularities including breath holding and hyperventilation occurred in approximately one third of our CDKL5 affected sample (32 %) compared with 15/20 females in the French clinical study [19]. In an Italian/Swedish case series of ten patients, respiratory dysrhythmia including forceful (*n* = 8) and apneustic breathing (*n* = 2) types was present in all the ten females [27]. In the same year, a Dutch study found out that two of the four CDKL5 affected children observed had central apnoeas when awake, preceded by recurrent episodic hyperventilation [24]. The differences between the findings of our study and these small case studies are not surprising; our larger study sample allows a better estimation of the prevalence of parent-reported breathing abnormalities. On the other hand ours was an international epidemiological study without the ability to measure autonomic and cardiorespiratory function as was used to identify the cardiorespiratory phenotype in those European patients [19, 24]. Therefore it is also feasible that autonomic abnormalities occur in the CDKL5 disorder without overt manifestations of breath holding and hyperventilation.

Lower respiratory tract infections including pneumonia were reported for over one fifth of the CDKL5 affected individuals. This is an important issue especially among the younger children that might relate to feeding and swallowing difficulties, aspiration or difficulties in expectorating chest secretions due to a poorly effective cough. Aspiration of food or fluids into the lungs was reported for nearly a quarter of the affected individuals. Over a quarter were also unable to cough up chest secretions, which might be a contributing risk factor for aspiration because coughing is a protective mechanism to prevent aspiration [49].

Approximately one third of individuals would have developed scoliosis by the age of 10 years. In comparison to other comorbidities, scoliosis was the least frequently observed. The occurrence of scoliosis has been rarely reported previously in the literature, possibly due to the younger age distributions in these studies. A study by Artuso et al. [17] reported absence of scoliosis in the nine CDKL5 positive females (age range 1.2 to 13 years) studied. The larger sample size of our study and comparatively wider age range of the participants allowed a better estimation of the occurrence of scoliosis in CDKL5 disorder because this is a comorbidity that develops over time.

Age can be considered as a risk factor for comorbidities such as epilepsy, GI and sleep problems, respiratory issues and scoliosis. The likelihood of each of these comorbidities increased with age except for the frequency of coughing during feeding. Younger children would be more prone to coughing as their small and narrow airways get obstructed easily [50]. Over time, some would have had gastrostomy insertions, which could explain the reduced symptoms of dysphagia in older children.

The influence of gender on the prevalence of various comorbidities was clearly evident. As generally reflected in our data, males were more vulnerable to various respiratory and sleep problems. X-inactivation (XCI) and different degrees of skewing in females may result in variable expression of CdKL5 protein, which may improve the expression of the phenotype in females. XCI is the transcriptional inactivation of one of the two X chromosomes in females to functionally equalize the gene dosage imbalance of X linked genes between XX females and XY males [51]. Our recent study on developmental milestones in this disorder, also using data from the ICDD, reported that males were more affected overall with more impaired development than females; this was consistent with our results [23].

Even though no significant relationship between mutation type and various comorbidities was found, there were consistencies in the distribution of comorbidity status by mutation group. For instance, there was some evidence that mutations associated with late truncations after aa781 may play a protective role against some GI problems, autonomic breathing irregularities and sleep problems compared to those with no function protein. Individuals with missense/in-frame mutations within the catalytic domain appeared to have higher odds of having any GI problems (constipation, reflux and air swallowing) and autonomic breathing irregularities (hyperventilation and breath-holding) when compared to those with no functional protein. We previously showed that late truncation mutations after aa781 were generally associated with a milder phenotype and had attained better gross motor, hand function and communication skills than those with other mutations [23]. Some genotype-phenotype relationships were also found in the French clinical series of 12 cases [20]. There patients with missense mutations in the catalytic domain (*n* = 4) had better walking and hand use ability and less frequent refractory epilepsy compared with those with mutations in the kinase domain and frameshift mutations in the C-terminal region (*n* = 7) who had a severe phenotype including infantile spasms, frequent refractory epilepsy, severe motor impairment and inability to walk.

There have been several studies [2, 32, 52] reporting on overlapping similarities between the clinical features and comorbidities of CDKL5 disorder and Rett syndrome. This is thought possibly to be due to the same signalling pathway shared by the CdKL5 and MeCP2 protein. Pini et al. [27] suggests that there might be a direct phosphorylation of MeCP2 protein by CdKL5 that has a kinase domain. Despite the apparent similarities in many of the clinical manifestations of the CDKL5 disorder and Rett syndrome, there are some differences in their occurrence and onset between the two disorders. Epilepsy is the hallmark feature of CDKL5 disorder with almost every case having at least some seizures. Epilepsy in CDKL5 disorder is characterized by complex partial seizures, infantile spasms, myoclonic and tonic seizures [17–19]. The onset of epilepsy is much earlier (1.4 months) in the CDKL*5* disorder than in Rett syndrome (4.9 years). As reflected in our time-to-event curve (Fig. 4), the risk of developing seizures was significantly higher in CDKL5 affected females than those with RTT during the first 20 years of age. In a study conducted on 685 females with Rett syndrome using the InterRett database, only one quarter of the females with Rett syndrome had seizures by the age of 3 years [53].

Gastrointestinal problems except for reflux were slightly less prevalent in CDKL5 affected females than Rett syndrome. Rett syndrome is generally associated with a high prevalence of gastrointestinal dysmotility, feeding difficulties and poor growth [6, 54, 55]. In a study by Motil et al. [54], gastrointestinal problems (91 %) and feeding problems (81 %) were highly prevalent with gastroesophageal reflux less prevalent. On the other hand, the incidence of gastrostomy insertion was found to be slightly higher in the CDKL5 disorder than Rett syndrome. A possible explanation could be due to the use of gastrostomy for purposes other than feeding difficulties in the CDKL5 disorder, especially for the administration of medications or the ketogenic diet needed to manage early onset and persistent seizures.

Sleep problems including night waking, screaming and laughing were more likely to occur in the CDKL5 disorder than Rett syndrome. The likelihood of experiencing a sleep problem in the CDKL5 disorder increased with age. The reason for this is not clear but it could relate to the persistent and refractory epilepsy. Our findings also suggest that autonomic dysfunction is less affected in CDKL5 disorder than in Rett syndrome. On the other hand, lower respiratory tract infections such as pneumonia, were a little more likely to occur in CDKL5 affected females than in those with Rett syndrome. This might be due to increased occurrence of reflux and feeding difficulties in CDKL5 disorder contributing to increased risk of aspiration.

Our study comprised the largest number of individuals with a confirmed pathogenic *CDKL5* mutation to date therefore enabling greater precision in our statistical estimates. This is the first time a CDKL5 specific questionnaire has been used to investigate the comorbidities of the disorder. Engagement of the consumer reference group in the development of the questionnaire also gives strength to this study. The ICDD is growing and in time will provide an even larger data repository for the better understanding of various aspects of the CDKL5 disorder. In addition, compared to the previous case series and case studies, the age range of the individuals in the CDKL5 registry is wide providing a better estimate of the prevalence and characteristics of comorbidities, particularly those that develop with time.

There were some limitations in this study that might impact the results. As the CDKL5 disorder questionnaire asks parents to provide information of their child’s condition since birth, there is a possibility for some recall error. To counter this, parents were asked to refer to their child’s medical records and diaries before the completion of questionnaire. Families did provide very extensive medical histories about their children but we acknowledge this was from a patient rather than a clinical perspective. Another limitation is that, a complete record of information was not available from every confirmed case, as parents may have completed different sections, resulting in varying denominators for various outcomes. Since the CDKL*5* disorder is a condition that requires expensive genetic testing for diagnosis, selection bias according to socioeconomic status may be occurring. Families from higher socioeconomic backgrounds are more likely to have the financial capacity or insurance to cover the costs of genetic testing, especially in countries such as USA where the socioeconomic gradient is marked. This may result in many individuals with a *CDKL5* mutation remaining undiagnosed and thus excluded from the registry. This is a cross sectional data collection, and longitudinal data collection similar to Rett syndrome [14] would be helpful to understand the progression and severity of the clinical features over time.

## Conclusion

This study provides a comprehensive picture of comorbidities in the CDKL5 disorder. Being the largest sample to date, the assumptions and results obtained gives a much more precise insight into the phenotypic expression of this disorder than was previously possible. Epilepsy, gastrointestinal and sleep problems were shown to be extremely prevalent, whilst scoliosis and respiratory problems were relatively less so. We have demonstrated that the impacts of daily feeding and sleeping difficulties in CDKL5 disorder both for the child and their family are issues of concern in urgent need of further clinical research. There is also a need to evaluate quality of life both for the child and their family and for this to be considered as an important outcome when investigating optimal strategies for managing the many complexities of this disorder. Finally it is heartening to know that the growing size of the CDKL5 registry will provide a valuable platform for further future study of genotypic-phenotypic relationships in this disorder.

## Abbreviations

AA: amino acid
ARSD: Australian Rett Syndrome Database
CDKL5: cyclin-dependent Kinase-like 5
CI: confidence interval
DA: disorders of arousal
DIMS: disorders of initiating and maintaining sleep
EEG: electroencephalography
ICDD: International CDKL5 disorder database
InterRett: International Rett syndrome Phenotype Database
IRR: incidence rate ratio
MECP2: methyl-CpG binding protein 2
OR: odds ratio
SDSC: sleep disturbance scale for children
SHY: sleep hyperhidrosis
SBD: sleep breathing disorders
SWTD: sleep wake transition disorder
RRR: relative risk ratio
